# Origin, variation, and selection of natural alleles controlling flowering and adaptation in wild and cultivated soybean

**DOI:** 10.1007/s11032-023-01382-4

**Published:** 2023-04-29

**Authors:** Zhihong Hou, Chao Fang, Baohui Liu, Hui Yang, Fanjiang Kong

**Affiliations:** Guangdong Key Laboratory of Plant Adaptation and Molecular Design, Guangzhou Key Laboratory of Crop Gene Editing, Innovative Center of Molecular Genetics and Evolution, School of Life Sciences, Guangzhou University, Guangzhou, 510006 China

**Keywords:** Wild soybean, Cultivated soybean, Photoperiodic flowering, Adaptation, Natural selection, Artificial selection

## Abstract

Soybean (*Glycine max*) is an economically important crop worldwide, serving as a major source of oil and protein for human consumption and animal feed. Cultivated soybean was domesticated from wild soybean (*Glycine soja*) which both species are highly sensitive to photoperiod and can grow over a wide geographical range. The extensive ecological adaptation of wild and cultivated soybean has been facilitated by a series of genes represented as quantitative trait loci (QTLs) that control photoperiodic flowering and maturation. Here, we review the molecular and genetic basis underlying the regulation of photoperiodic flowering in soybean. Soybean has experienced both natural and artificial selection during adaptation to different latitudes, resulting in differential molecular and evolutionary mechanisms between wild and cultivated soybean. The in-depth study of natural and artificial selection for the photoperiodic adaptability of wild and cultivated soybean provides an important theoretical and practical basis for enhancing soybean adaptability and yield via molecular breeding. In addition, we discuss the possible origin of wild soybean, current challenges, and future research directions in this important topic.

## Introduction

Modern crops are domesticated from their wild relatives over long periods of time. Crop domestication refers to the morphological and physiological changes that cultivated crops and their wild ancestors have undergone through conscious or unconscious artificial selection and the continuous accumulation of favorable characteristics required by humans. Therefore, crop domestication is the long-term result of both natural and artificial selection. Domestication leads to drastic changes in morphology and physiology between domesticated crops and their wild ancestors, which is known as “domestication syndrome” (Hammer 1984; Doebley et al. 2006). As humans spread these domesticated plants to a wider geographical area, the sizes of plant populations with allelic variations suitable for different environments gradually selected and expanded due to intentional planting by farmers.

The annual plant wild soybean (*Glycine soja* Sieb. & Zucc.) is the wild ancestor of cultivated soybean (*Glycine max* (L.) Merr.). Cultivated soybean is thought to have been domesticated from its wild soybean ancestor more than 5000 years ago in China, after which it spread worldwide (Hymowitz 1970; Carter et al. 2004; Li et al. 2008). Cultivated soybean has become one of the most economically important oil and protein crops worldwide, providing more than one-quarter of the world’s protein for human food and animal feed (Graham and Vance 2003; Hartman et al. 2011). However, due to long-term domestication and improvement, less than 50% of variations from wild soybean have been selected, and many important genes/alleles related to environmental adaptation have been lost, which greatly hinders the further improvement of the yield and quality of cultivated soybean (Zhou et al. 2015; Kofsky et al. 2018).

The growth and development including flowering of crops are regulated by photoperiod. Photoperiodic responses play important roles in crop introduction and acclimation. Most plants have obvious photoperiodic flowering responses. Flowering time determines the geographical adaptability of crops and affects final crop yields. Flowering time is an important ecological indicator of the photoperiodic response in soybean and is also a critical agronomic character to influence grain yield, seed quality, and adaptability. Both wild and cultivated soybean are short-day plants and are highly sensitive to photoperiod, making the growth of individual wild soybean accessions or individual soybean varieties generally limited to a small range of latitudes (Watanabe et al. 2012). Compared to cultivated soybean, the flowering and maturity periods of wild soybean are very late. During domestication, the photoperiod sensitivity of cultivated soybean is significantly reduced to fit the early and synchronized harvest and maximize the grain yield in the cultivation regime (Lu et al. 2020).

Wild soybean is distributed across a fair broad geographical range (24–53°N, 97–143°E); likewise, cultivated soybean is also planted in a wider range of latitudes across the world, from 53°N to 35°S (Qiu et al. 2013; Li et al. 2014; Zhang et al. 2020). The wide distribution of cultivated soybean and its wild ancestors is controlled by many maturity genes, as reflected by quantitative trait loci (QTLs) controlling photoperiodic flowering and maturity. Several major flowering and maturity loci have been identified and functionally characterized in soybean, including maturity genes, long-juvenile (LJ) genes, and several QTLs (Lin et al. 2021a, 2021b; Hou et al. 2022; Du et al. 2023; Liang and Tian 2023; Wang et al. 2023a). Variations or natural alleles of these genes or QTLs controlling photoperiodic flowering and maturation have been artificially or naturally selected at different latitudes, allowing soybean to adapt to wide latitudes. Current findings suggest that genetic and molecular mechanisms evolved independently in both wild and cultivated soybean when these plants adapted to high latitudes (long-day conditions) or low latitudes (short-day conditions). In addition, the evolutionary adaptation mechanisms of the wild by natural selection and cultivated soybean by artificial selection are quite different. In this review, we summarize and discuss distribution, possible origin, and photoperiodic flowering control networks of wild and cultivated soybean, with a focus on the natural selection and artificial selection of genes related to photoperiod in wild and cultivated soybean adapted to different latitudes.

## Origin and geographical distribution of wild and cultivated soybean

Wild soybean is a temperate species found in East Asia north of the Tropic of Cancer, including China, Korea, Japan, and the far eastern regions of Russia (Wilson 2008; Zhou et al. 2015). Wild soybean is most widely distributed in China where it is present in all provinces except Qinghai, Xinjiang, and Hainan provinces (Li 1994; Dong et al. 2001). In China, the distribution area of wild soybean ranges from Yisiken (53° N) in Tahe County, Heilongjiang Province in the north, Xiangzhou (24° N) in Guangxi Province, and Yingde (24° 10′ N) in Guangdong Province in the south, Fuyuan (134° 20′ E) in Heilongjiang Province in the east, to Shangchayu District (97° E) in Zayu County, Tibet, in the west. The upper limit of the altitude distribution of wild soybean is 1300 m above sea level in Northeast China, 1500–1700 m above sea level in the Yellow River and Yangtze River valleys, and 2250 m above sea level in Tibet. The highest point of distribution of wild soybean in China is Ninglang County, Yunnan Province at 2650 m above sea level (Zhuang 1999; Dong et al. 2001).

It is generally accepted that the cultivated soybean was domesticated from its wild ancestor in the Huang-Huai-Hai region of China between 32° and 40° N, resulting in regional local landraces, and was further crossed and selected during breeding to generate modern cultivated soybean varieties (Hymowitz 1970; Carter et al. 2004; Li et al. 2008; Wilson 2008). As China represents the origin of cultivated soybean, almost all soybeans grown in other regions of the world have directly or indirectly spread from China. Soybean may have been introduced to Korea, Japan, and South Asia approximately 2000 years ago, to Europe and North America in the middle of the eighteenth century, and to Central and South America in the first half of the twentieth century (Wilson 2008).

The long history of domestication, cultivation, and breeding has narrowed the genetic basis of cultivated soybean and has restricted the further improvement of the yield and quality. By contrast, wild soybeans, which inhabit a wide geographical area in East Asia, show extensive genetic variation in pest and disease resistance genes and other useful agricultural and ecological characteristics. Understanding the origin of wild soybean could shed light on the mechanism of genetic penetration of wild soybean into cultivated soybean, provide theoretical guidance for the innovation and improvement of soybean germplasm, and clarify the basic laws of soybean variety improvement. However, to date, since few studies have focused on the origin and evolutionary pattern of wild soybean, the origin of wild soybean is still unresolved.

## Molecular mechanisms of photoperiodic responses in soybean

Flowering time is a key agronomic trait with significant effects on plant yield and quality (Lin et al. 2021b). Flowering time is modulated by a combination of environmental and endogenous signals, one of the most important of which is photoperiod (Song et al. 2015). In recent years, researchers have identified many major maturity genes and QTLs and have uncovered their functions and molecular mechanisms in the regulation of photoperiodic flowering in soybean under long- and short-day conditions.

In soybean, the classical maturity loci *E1* to *E11*, the LJ locus *J*, *FLOWERING TIME 2a* (*FT2a*, also named *LJ16.1*), *FT5a* (also named *LJ16.2*), and several other QTLs including *Time of Flowering 4* (*Tof4*), *Tof5*, *Tof11* (also named *Growth period 11* [*Gp11*]), *Tof12* (also named *Gp12/qFT12-1*), *Tof16*, *Tof18*, and QTL near *E1* (*QNE1*) have been identified in segregating populations derived from crosses between cultivars with contrasting phenotypes (Bernard 1971; Buzzell 1971; Buzzell and Voldeng 1980; McBlain and Bernard 1987; Bonato and Vello 1999; Cober and Voldeng 2001; Cober et al. 2010; Kong et al. 2014; Samanfar et al. 2017; Wang et al. 2019; Ray et al. 1995; Dong et al. 2021, 2022a, 2023; Lu et al. 2017, 2020; Li et al. 2021; Kou et al. 2022; Xia et al. 2022). The dominant alleles of *E1*, *E2* (as homologue of *GIGANTEA* [*GIa*]), *E3* (homologue of *PHYTOCHROME A3*, *PHYA3*), *E4* (*PHYA2*), *E7*, *E8*, *E10* (also named *FT4*), *Tof4* (*E1* like 1a, *E1la*), *Tof11* (*PSEUDO-RESPONSE REGULATOR 3a*, *PRR3a*), and *Tof12* (*PRR3b*) delay flowering, whereas the dominant alleles of *E6/J* (*EARLY FLOWERING 3*, *ELF3*), *E9* (also named *LJ16.1*, *FT2a*), *E11*, *LJ16.2* (*FT5a*), *Tof5* (*FRUITFULL 2a*, *FUL2a*), *Tof16* (*LATE ELONGATED HYPOCOTYL 1a*, *LHY1a*), *Tof18* (*SUPPRESSOR OF OVEREXPRESSION OF CO 1a*, *SOC1a*), and *QNE1* promote flowering (Dong et al. 2023; Du et al. 2023; Hou et al. 2022; Lin et al. 2021a, 2021b, 2022; Wang et al. 2023a, 2023b). Besides the above loci/genes, some other important genes have been characterized through reverse-genetic approaches and have been shown to be involved in the photoperiodic control of flowering time, such as the circadian clock genes *LUX ARRYTHMO* (*LUX1*) and *LUX2* (Bu et al. 2021) and *NIGHT LIGHT-INDUCIBLE AND CLOCK-REGULATED 2* (*LNK2*) (Li et al. 2020b), *CONSTANS-like* (*COL1a*, *COL1b*, *COL2a*, *COL2b*) (Cao et al. 2015; Wu et al. 2019), *TARGET OF EAT 1* (*TOE4a*) (Zhao et al. 2015), *RELATED TO ABI3/VP1* (*RAV*) (Wang et al. 2021c), *GUANYLATE-BINDING PROTEIN 1* (*GBP1*) (Zhao et al. 2018), and *AGAMOUS*-*like1* (*AGL1*) (Zeng et al. 2018). These findings shed light on the genetic mechanisms of soybean adaptability and provide valuable genetic resources for the molecular design breeding of soybean cultivars with high yields.

Unlike the model species Arabidopsis (*Arabidopsis thaliana*) (Shim et al. 2017), the photoperiod flowering pathways in soybean is centered on the legume-specific B3-like transcriptional repressor E1 (Xia et al. 2012; Xu et al. 2015). E1 integrates upstream light receptors (E3 and E4) and circadian clock (E2, J, LUX1, LUX2, Tof11, Tof12, and Tof16) signals to downstream genes to control photoperiodic flowering (Du et al. 2023; Hou et al. 2022; Lin et al. 2021b). Tof11 and Tof12 promote *E1* expression by repressing the circadian clock gene *LHY* under long-day conditions (Lu et al. 2020). The circadian evening complex (EC) in soybean, comprising J, LUX1, and LUX2, suppresses the expression of *E1* and its two homologs, *E1La* and *E1Lb*, by directly binding to their promoters (Bu et al. 2021; Lu et al. 2017). *E2*, an ortholog of Arabidopsis *GI*, is genetically dependent on *E1* and *E1L*s. E2 can form homodimers or heterodimers with other two E2-like proteins to induce the transcription of *E1* and *E1* homologs to inhibit flowering (Wang et al. 2023b). The phytochrome A homologs E3 and E4 induce *E1* and *E1L* expression via multifaceted approaches: (1) E3 and E4 positively regulate the expression of *Tof11* and *Tof12* to affect E1 activity (Lu et al. 2020); (2) E3 and E4 interact with and degrade LUX proteins to relieve the inhibition of *E1* by the EC (Lin et al. 2022); (3) E3 and E4 directly associate with E1 and enhance its stability at the posttranscriptional level (Lin et al. 2022).

**Fig. 1:**
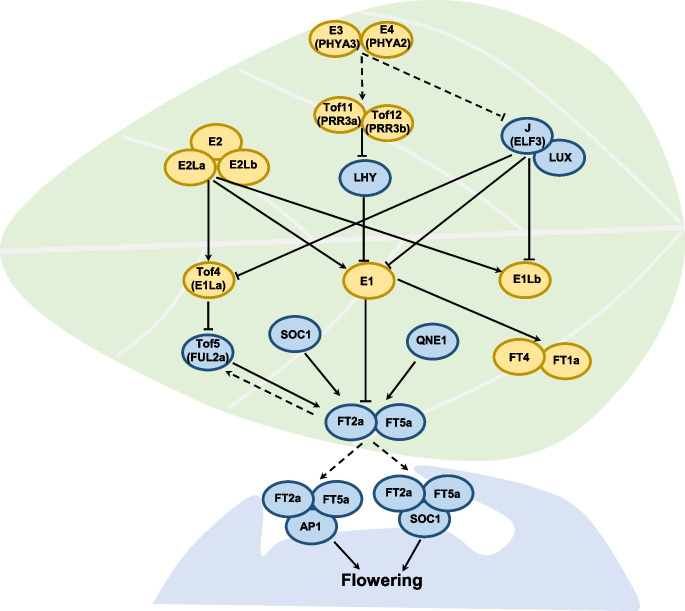
Photoperiodic flowering regulatory mechanisms in soybean. Yellow oval represents flowering repressors; blue oval indicates flowering promoters. The solid and dotted lines represent direct and indirect regulation, respectively. Arrow means activation, while T-shape symbol means suppression

As the central hub in soybean photoperiod flowering, E1 then transfers these signals to the florigen genes *FT*s as output (Du et al. 2023; Hou et al. 2022; Lin et al. 2021b). E1 confers late flowering, mainly by inhibiting the expression of *FT2a* and *FT5a* (Kong et al. 2010; Nan et al. 2014; Thakare et al. 2011; Xia et al. 2012; Xu et al. 2015), as well as inducing the expression of the flowering repressor genes *FT1a* and *FT4* (Zhai et al. 2014). *Tof5* links *E1* to *FT2a* and *FT5a*: E1 directly represses *Tof5*, and Tof5 directly activates *FT2a* and *FT5a* to promote flowering (Dong et al. 2022a). A recent study revealed that Tof4/E1La has a function similar to E1, which directly associates with the promoters of *Tof5*, *FT2a*, and *FT5a* to suppress their transcription (Dong et al. 2023). In addition, Tof18 directly activates the expression of *FT2a* and *FT5a* in leaves (Kou et al. 2022), and QNE1 can form protein complexes with FT2a and FT5a (Xia et al. 2022). Furthermore, FT2a and FT5a interact with FDLs (FLOWERING LOCUS D-LIKE) to induce the expression of the floral meristem identity gene *APETALA1* (*AP1*) to promote flowering (Chen et al. 2020; Li et al. 2021; Nan et al. 2014; Yue et al. 2021). Based on these findings, various molecular regulatory networks of photoperiodic flowering modules have been proposed in soybean, including E3/E4-Tof11/Tof12-LHY-E1-FT, E3/E4-EC-E1-FT, E3/E4-E1-FT, E2-E1-FT, and FT/FD-AP1 (Fig. 1). Interestingly, the E3/E4-E1-FT pathway was recently shown to participate in delaying flowering mediated by high temperature (35 °C) in soybean (Tang et al. 2023). The above photoperiod genes constitute a highly complex photoperiod-regulated flowering network. Further identification of new genes/alleles related to this network should shed light on the selection of photoperiod-regulated genes during domestication and variation and provide a genetic basis for the adaptation of wild and cultivated soybean to the broad ecological environment.

## Natural variation and artificial selection for the photoperiodic adaptation of wild and cultivated soybean

### Mechanisms of cultivated soybean adaptation

Cultivated soybean originated in central China around the Huang-Huai Valley, a mid-latitude temperate region. Subsequently, cultivated soybean underwent dissemination and adaptation following two directions, northward to high latitudes and southward to low latitudes, spreading to all parts of the world, and it is now widely planted worldwide. As soybean is a typical photoperiod-sensitive plant, the growth of individual cultivars is generally limited to a narrow range of latitudes (Watanabe et al. 2012). However, modern cultivated soybean is widely adapted to ecological environments at different latitudes. How did its extensive regional adaptability occur? What are the underlying genetic and molecular mechanisms?

Some important flowering inhibitor genes such as *E1*, *E3*, *E4*, *Tof5*, *Tof11*, and *Tof12* accumulated sequence polymorphisms during the adaptation of cultivated soybean northward to high latitudes under longer daylengths. These genetic changes reduced photoperiod sensitivity to produce early flowering. Variation leading to early flowering was artificially selected, allowing cultivated soybean to adapt to high-latitude areas (Abe et al. 2003; Cober et al. 1996; Liu and Abe 2010; Xu et al. 2013; Dong et al. 2022a; Li et al. 2019a, 2019b, 2020a; Wang et al. 2020; Lu et al. 2020). On the contrary, during the adaptation of soybean to low-latitude regions with shorter daylengths in the south, the flowering-promoting factors *J*, *Tof16*, *Tof18*, *FT2a*, and *FT5a* were genetically impaired. These new polymorphisms extended the growth period of cultivated soybean under short-day conditions at low latitudes, thus improving the adaptability and increasing the yield of soybean (Lu et al. 2017; Ray et al. 1995; Yue et al. 2017; Fang et al. 2021; Li et al. 2021; Dong et al. 2021; Kou et al. 2022).

#### Adaptation of cultivated soybean to high latitudes

When cultivated soybean is grown in high-latitude regions, the days are longer, flowering time is delayed, and the plant cannot mature properly before the early frost, leading to a substantial decline in yields. Therefore, cultivated soybean must reduce the photoperiod sensitivity to be able to flower and mature early under long-day conditions. Flowering and maturing at a suitable time guarantee high soybean yields in high-latitude regions, which is mainly achieved by reducing the accumulation of photoperiod-sensitive alleles. Analyzing the natural and artificial selection of these alleles and their distribution can reflect their importance to the adaptability of cultivated soybean.

The classical maturity loci *E1*, *E3*, and *E4* play important roles in regulating photoperiodic sensitivity and adaptability to high latitudes (Xu et al. 2013). *E1*, *E3*, and *E4* have a variety of loss-of-function mutations. Different combinations of these mutations determine the ecological adaptability of soybeans to middle and high latitudes (Xu et al. 2013) and have been widely used in high-latitude breeding (Jiang et al. 2014; Cao et al. 2017). Functional types *E1*, *E3*, and *E4* alleles delay flowering and maturity. Four major *E1* alleles have been identified in different soybean cultivars: *E1*, *e1*^*as*^, *e1*^*fs*^, and *e1*^*nl*^. Among them, *e1*^*fs*^ and *e1*^*nl*^ are functionally deficient, leading to very early flowering and maturity. By contrast, *e1*^*as*^ is a weak mutant allele with an effect intermediate between that of the *E1* genotype and the functionally deficient alleles (Xia et al. 2012; Xu et al. 2013; Tsubokura et al. 2014). Six alleles of the *E3* gene have been identified in soybean: *E3-Mi*, *E3-Ha*, *e3-tr*, *e3-Mo*, *e3-ns*, and *e3-fs* (Watanabe et al. 2009; Xu et al. 2013; Li et al. 2014). There are five other types of *E4* alleles: *e4-SORE1*, *e4-kam*, *e4-kes*, e*4-oto*, and *e4-tsu*. Among *E1*, *E3*, and *E4* loci, the most common genotype of photoperiod-insensitive varieties is *e3 e4*, accounting for 70% of all genotypes grown at high latitudes. The second most common genotype of photoperiod-insensitive varieties comprises recessive *e1* and any type of *e3* and *e4* allele, such as *e1 e3 E4* or *e1 E3 e4*. The third genotype, *e1*^*as*^ *e3 E4*, is found in light-insensitive varieties (Xu et al. 2013). These three genotypes reduce the sensitivity of soybean to long-day conditions to different extents.

Two key loci that control the flowering stage of soybean, *Gp11/Tof11* and *qFT12-1/Gp12/Tof12*, play important roles in the domestication and adaptability of cultivated soybean to high-latitude regions (Liu et al. 2018; Li et al. 2019a, 2019b, 2020a; Lu et al. 2020; Wang et al. 2020). These two loci encode PRR3a and PRR3b, which are homologs of Arabidopsis PRR3 (Li et al. 2019a, 2019b, 2020a; Lu et al. 2020; Wang et al. 2020). *Tof11* and *Tof12* play independent roles in regulating flowering and maturity, but there is some functional redundancy (Lu et al. 2020). *Tof11* and *Tof12* have undergone gradual variation and artificial selection during evolution (Lu et al. 2020). Almost all cultivated soybeans carry *tof12*, a nonfunctional allele, indicating that this allele has undergone strong artificial selection during domestication and early evolution (Li et al. 2019b, 2020a; Lu et al. 2020; Wang et al. 2020). A study analyzing variation in the *Tof12* coding sequences of 1295 soybean materials (including wild soybean, landraces, and cultivated soybean) uncovered 25 *Tof12* haplotypes, including four nonfunctional alleles (*tof12-1* to *tof12-4*). The null allele *tof12-1*, which confers early flowering, is the most common, as it was detected in all soybean cultivars (532/532) and most landraces (406/450), indicating that this allele is favored in cultivated soybean (landraces and cultivars) and was intensively used during early soybean breeding. The other three *tof12* (*tof12-2* to *tof12-4*) nonfunctional alleles are rare in wild soybean and landraces which are not incorporated in breeding yet. Therefore, the *tof12-1* mutation played a central role in the domestication and adaptation of soybean (Lu et al. 2020).

Among the 1295 soybean materials examined by Lu et al. (2020), 11 nonfunctional alleles of *Tof11* were identified: *tof11-1* to *tof11-11*. The early flowering null allele *tof11-1* is the most abundant, accounting for 507/552 cultivated soybean lines and 192/520 landraces. Nonfunctional alleles of *Tof11* have independently appeared many times in three pedigrees of wild soybean, landraces, and cultivated soybean, but only the *tof11-1* allele is widely selected in landraces and cultivars. The functional deletion mutation of *tof11-1* was selected again in the *tof12-1* genetic background in a stepwise manner, which further shortened the flowering and growth periods of cultivated soybean and improved the adaptability of cultivated soybean to high latitudes (Lu et al. 2020). Analysis of the *Tof11*, *Tof12*, and *E1* loci suggests that these three loci have made sequential contributions to soybean domestication and its expansion to high-latitude regions. In addition, the selection of the *tof12* allele is in parallel and similar with the three most important domestication genes: *Shat1-5*, *Hs1-1*, and *G* which control the pod-falling, seed hardness, and dormancy traits. The genomic selection features of *tof12-1* indicate the selection of photoperiod insensitivity is the key event of early soybean domestication. It therefore is the clear result that early phenology with reduced photoperiod sensitivity is considered the classical crop domestication trait (Lu et al. 2020; Gong 2020).

Recently, another two genetic loci *Tof5* (Dong et al. 2022a) and *Tof18* (Kou et al. 2022) are identified and classified to play important roles in soybean adaptation to high-latitude regions. The early flowering allele of *Tof5*^*H1*^ is frequently selected in the accessions of cultivated soybean in high latitudes suggesting that the *Tof5*^*H1*^ allele experienced artificial selection and promoted the adaptability of cultivated soybean to high latitudes (Dong et al. 2022a). *Tof18* encodes *SOC1a*, which promotes flowering in soybean and affects the number of main stem nodes and yield under both long and short-day conditions. By analyzing the nucleotide polymorphism of *Tof18*/*SOC1a* in 349 soybean materials, *Tof18* was divided into two types: *Tof18*^*A*^ and *Tof18*^*G*^, an SNP variation in the promoter of *SOC1a* which controls the transcriptional level of *Tof18*/*SOC1a*. Compared to the *Tof18*^*A*^ allele, plants harboring the *Tof18*^*G*^ allele flowered earlier and showed significantly reduced plant height, branch number, and total grain number. The *Tof18*^*G*^ allele therefore promotes the adaptability of soybean to high-latitude areas (Kou et al. 2022). Identification and characterization of additional novel loci/genes will further improve the understanding on the genetic mechanism underlying high latitude adaptation in soybean.

#### Adaptation of cultivated soybean to low latitudes

Temperate soybean cultivars when grown under low latitudes and short-day conditions flower very early and result in very shorter plants and extremely low grain yield. The LJ trait was incorporated into the soybean cultivars in the 1970s in low latitudes (Hartwig and Kiihl 1979) which delays flowering under short-day, high-temperature conditions, allowing the plant to obtain sufficient nutrients during growth, thereby improving yields. Therefore, the introduction of the LJ trait allowed cultivated soybeans originating from the temperate Huang-Huai-Hai region to adapt to the ecological environment of tropical low-latitude regions (Hartwig and Kiihl 1979; Spehar 1995). The discovery and application of the LJ trait have made low-latitude areas that were previously not suitable for soybean cultivation rapidly develop into major soybean production areas, thus altering worldwide soybean production and trade. Brazil has become the world’s largest soybean producer. Indeed, the soybean output from low-latitude regions has exceeded half the total soybean output worldwide. The genetic locus controlling the LJ trait has been gradually defined.

*J* is the key locus that controls the LJ traits of soybean and is crucial for the adaptive evolution of soybean (Yue et al. 2017; Lu et al. 2017; Dong et al. 2021). *J* is a homologous gene of Arabidopsis *ELF3* and encodes an evening complex component of the core circadian clock (Lu et al. 2017; Fang et al. 2020; Bu et al. 2021). The mutation of *J* can delay flowering in soybean under low latitudes and short-day conditions and increase the yield by 30 ~ 50% compared to the wild-type allele (Lu et al. 2017). In total, at least 8 loss-of-function variants of *j* are identified and have played critical roles in the adaptation of soybean to low-latitude areas and promotion and production in these areas (Lu et al. 2017).

*E6* is another classical locus for soybean adaptation into low latitudes. The mutational allele *e6*^*PG*^ harbors a *Ty1/Copia*-like retrotransposon and is identified as a *J* allele and named *j-9*, extending the number of* J* alleles to nine (Fang et al. 2021). Dong et al. (2021) also identified two nonfunctional alleles of *J* named *j-10* and *j-11*. *j-11* is a weak functional allele that originated in wild soybeans in the Huang-Huai-Hai region of China and was subsequently transferred to cultivated varieties during domestication; this allele was strongly selected in soybean varieties grown at low latitudes. Therefore, during the adaptation of soybean from temperate zones to low-latitude regions, the weak functional allele *j-11* was selected first, which improved soybean yields in low-latitude regions. As soybeans continued to adapt to the tropics, *j-11* was unable to meet the demands for higher yields. Therefore, the variants *j-1*, *j-3*, *j-6*, and *j-10* continued to occur in the *j-11* genetic background, which facilitated plant adaptation to the tropical environment and improved yields. These findings point to the gradual selection of *j-11* followed by *j-1*, *j-3*, *j-6*, and *j-10* (Dong et al. 2021).

Another important gene of the LJ trait, *Tof16*, has also played an important role in plant adaption to low latitudes (Dong et al. 2021). *Tof16* encodes the circadian clock component *LHY1a*. *Tof16* delays flowering and improves yields in low-latitude regions. Four loss-of-function alleles of *Tof16* were identified in 1624 resequenced soybean accessions: *tof16-1* to *tof16-4*. Among them, *tof16-2* is a weak functional deletion allele that originated in wild soybean in the Huang-Huai-Hai region and was transferred to cultivated varieties during domestication. The variants *tof16-1* and *tof16-4* primarily occurred in soybean varieties in Brazil in the genetic background of *tof16-2*. During the adaptation of soybean from temperate to low-latitude regions, the weak functional allele *tof16-2* was selected first, which improved yields in low-latitude regions. As soybean continued to adapt to tropical areas, *tof16-2* no longer met the demands for high yields. Therefore, in the *tof16-2* genetic background, the variants *tof16-1* and *tof16-4* continued to occur, which facilitated plant adaptation to the tropical environment and improved yields. In summary, there were also gradual selections between *tof16-2*, *tof16-1*, and *tof16-4* (Dong et al. 2021). The accessions from different regions possess distinct alleles of *Tof16* or *J* suggesting that the selection of natural variants at *tof16* and *j* might have occurred independently. In addition, genomic analysis of soybean varieties from low-latitude tropical regions found that 80% of the varieties contained different variants at the *Tof16* or *J* locus, indicating that natural variation at the *Tof16* or *J* locus was the main genetic basis for the adaptation of cultivated soybean to tropical regions (Dong et al. 2021).

However, variation of the *J*/*E6* and *Tof16* genes alone cannot fully explain the genetic basis for the adaptation of cultivated soybean to low-latitude areas. The *FT* homologs *FT2a* and *FT5a*, overlapping with different QTLs conferring the LJ trait in soybean, also played an important role in the adaptation of soybean to tropical regions (Cai et al. 2020; Li et al. 2021). Variations at *FT2a* and *FT5a* can delay the flowering and maturity of soybean under short-day conditions. The *ft2a* and *ft5a* single mutants show a strong genetic compensation response, with a relatively small delay in flowering time, whereas *ft2a ft5a* double mutants do not exhibit this compensation response, instead displaying an enhanced LJ phenotype and producing higher yields under short-day conditions in low-latitude areas (Li et al. 2021). Sequencing analysis of a soybean population grown at low latitudes showed that the variants of *FT2a* and *FT5a* have different geographical origins and played different roles in the spread of soybean to low-latitude tropical regions, uncovering a new regulatory pathway independent of the classical LJ locus *J* (Li et al. 2021). Moreover, the *Tof18*^*A*^ allele, which confers late flowering, also promotes the adaptability of soybean to low-latitude areas (Kou et al. 2022). These findings provide a new strategy for improving the adaptability and yield of soybean in tropical environments. Integrating the natural variation of flowering genes to enhance adaptation to the local environment and to improve yields provides an important perspective for molecular breeding.

### Mechanisms of wild soybean adaptation

Crop ancestors usually have rich allelic diversity, most of which was lost during crop domestication (Zhou et al. 2015). Natural variants from wild ancestors can be introduced into modern crops through breeding to improve plant adaptability and yield. The discovery of genes related to the geographical adaptability of wild soybean will help improve the flowering mechanism of wild soybean and promote the improvement of cultivated soybean. However, few studies have explored the genetic basis of wild soybean adaptability; only two investigations have thus far reported on the adaptability of wild soybean to high latitudes.

*Tof5*^*H2*^, a gain-of-function allele of *Tof5*, was the first allele shown to be related to the adaptability of wild soybean to high latitudes (Dong et al. 2022a). Analysis of a population of 1667 wild soybean accessions, landraces, and cultivated soybean showed that the *Tof5*^*H2*^ allele only existed in wild soybean, with no *Tof5*^*H2*^ allele identified in landraces or cultivars. Therefore, the *Tof5*^*H2*^ allele may have been lost during soybean domestication. The *Tof5*^*H2*^ allelic variant was naturally selected, thus contributing to the adaptation of wild soybean to high-latitude areas (Dong et al. 2022a). In addition, the extent of natural variation at the key genes *E1*, *E3*, and *E4*, which determine the photoperiod sensitivity of cultivated soybean, was analyzed in 257 wild soybeans. Loss-of-function mutations of *E1* and *E4* were not detected in these 257 wild soybeans, while the loss-of-function allele of *E3* was only found in accessions from northern China, demonstrating that allelic variant of *E3* also participated in the adaptation of wild soybean to high latitudes. This finding suggests that the genetic basis of the adaptation of wild and cultivated soybean to high-latitude regions might be different (Dong et al. 2022a).

*Tof4* is a recently discovered locus that regulates flowering in wild soybean (Dong et al. 2023). *Tof4* inhibits flowering to enhance the adaptability of wild soybean to high latitudes. The *Tof4* locus was shown to harbor the *E1La* gene via population genetics and stable transformation of soybean. Under long-day conditions, the partial functional deletion allele variation (*tof4-1*) of *Tof4* significantly promoted flowering and improved the adaptability of wild soybean to high latitudes. Tof4 binds to the promoters of *FT2a*, *FT5a*, and *Tof5* to inhibit their transcription under long-day conditions and improves the adaptability of wild soybean to high latitudes (Dong et al. 2023).

Analysis of *Tof4* genotypes in 2387 sequenced wild and cultivated soybean accessions revealed five haplotypes for *Tof4*: *Tof4*^*H1*^ to *Tof4*^*H5*^. *Tof4*^*H2*^ and *Tof4*^*H3*^ are weak functional deletion alleles, while *Tof4*^*H4*^ and *Tof4*^*H5*^ are fully functional. *Tof4*^*H2*^ corresponds to *tof4-1*, while *Tof4*^*H3*^ is also named *tof4-2*. The two weak functional alleles of *Tof4*, *tof4-1*, and *tof4-2*, were present in 32.9% of the wild soybean materials. However, only 0.35% (3/857) of the cultivated soybean materials contained the *tof4-1* allele, which may have arisen by natural or artificial introgression of wild soybean, suggesting that the weak functional alleles *tof4-1* and *tof4-2* are lost during soybean domestication. In addition, an analysis of 441 wild soybean materials from different latitudes in China found that *tof4-1* and *tof4-2* only existed in wild soybean from northeast China, indicating that *tof4-1* and *tof4-2* were strongly selected during the adaptation of wild soybean to high-latitude environments. By contrast, 71.5% of the wild soybeans contained either *tof4* or *Tof5*^*H2*^, indicating that the natural variation of these two loci was the main genetic basis for the adaptation of wild soybean to high latitudes. These findings provide different insights for enhancing the high-latitude adaptability of cultivated soybean and cultivating high-quality, high-yielding soybean varieties. Almost no cultivated soybean line carries a mutant *tof4* or *Tof5*^*H2*^ allele. Therefore, introducing the *tof4-1* and *Tof5*^*H2*^ alleles into modern soybeans will be an effective way to obtain early-maturing soybeans and improve productivity in high-latitude regions (Dong et al. 2023).

### The possible origin of wild soybean

Wild soybean is distributed across a wide range of latitude in East Asia, which includes China, Japan, Korea, and Russia, and has adapted to a variety of ecological conditions (Qiu et al. 2013; Li et al. 2014). To date, few studies have focused on the adaptability of wild soybean to different latitudes. One important reason for this fact is that the origin of wild soybean has not been resolved. Photoperiod has a great influence on the geographical distribution of plants. Therefore, to explore the origin of wild soybean, the photoperiodic response characteristics of this plant should first be examined, as the photoperiodic responses of plants might be largely determined by the origin and the region where they first adapt. The phylogenetic process of wild plants is the outcome of long-term adaptation to their ecological environment. Long-day plants generally originated from temperate regions with longer daylength (high latitudes), such as pea (*Pisum sativum*), wheat (*Triticum aestivum*), and barley (*Hordeum vulgare*). While short-day plants usually originated from low tropical latitudes, such as common bean (*Phaseolus vulgaris*), rice (*Oryza sativa*), and maize (*Zea mays*) (Brambilla et al. 2017). The pea is a cool-season crop of higher latitudes and derives from the Middle East (Jing et al. 2010). Wheat and barley are also typical long-day crops that originated in western Asia (Jones et al. 2008; Haas et al. 2019). Short-day crops include common bean and maize, which were originated at relatively low latitudes. Common bean was independently domesticated in Andean South America and Mexico (Mamidi et al. 2013; Schmutz et al. 2014; González et al. 2021). Maize was domesticated from its wild ancestor teosinte (*Zea mays ssp. Parviglumis*), which originated in the Balsas region of southwestern Mexico (Matsuoka et al. 2002; Minow et al. 2018). Since soybean is a typical short-day plant, it should have been originated in tropical or subtropical areas. Therefore, wild soybean likely originated from a low-latitude area. In addition, wild soybean does not require vernalization or overwintering in order to flower. Therefore, it is likely that this short-day crop did not originate in the middle or high latitudes.

**Fig. 2:**
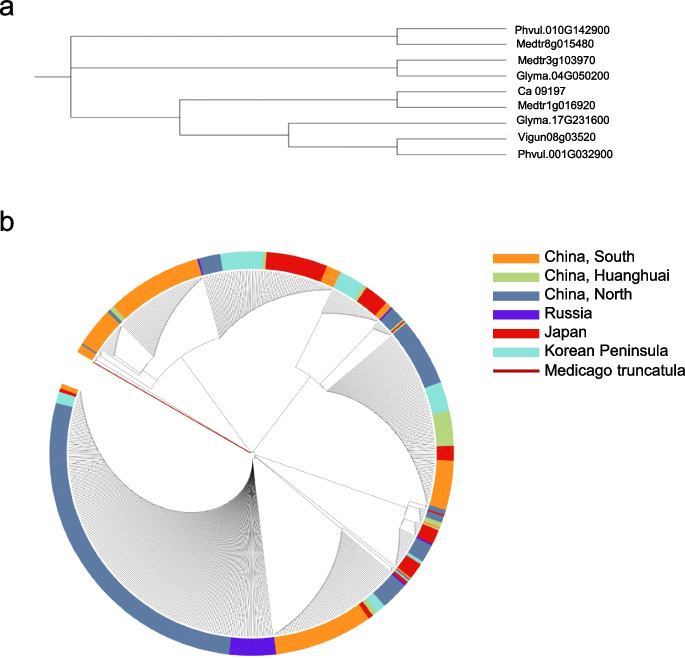
The origin of wild soybean deduced from the developmental tree of the J gene. **a** A phylogenetic tree of *ELF3* genes in *Glycine max*, *Phaseolus vulgaris*, *Vigna unguiculata*, *Ceratopteris richardii*, and *Medicago truncatula*. **b** A phylogenetic tree of *J* gene of wild soybeans from Southern China, Huanghuai-China, Northern China, Russia, Japan, and Korean Peninsula and the homologous gene of J in *Medicago truncatula*, the *ELF3* of *Medicago truncatula*, was defined as outgroup. The phylogenetic tree was inferred by using the maximum likelihood method and JTT matrix-based mode

To explore the origin of wild soybean, we constructed a phylogenetic tree using the *ELF3/J* gene (which is important for plant adaptation to low latitudes) about *Glycine max*, *Phaseolus vulgaris*, *Vigna unguiculata*, *Ceratopteris richardii*, and *Medicago truncatula*. We found that *ELF3* of *Glycine max* is closer and more similar to *Medicago truncatula* compared to the other accessions (Fig. 2a). Furthermore, we analyzed the phylogenetic tree of *ELF3* gene in 732 wild soybean germplasm resources that were described in a previous study (Lu et al. 2020; Dong et al. 2022a, 2023; Kou et al. 2022) and we have preserved collecting from Southern China, Huanghuai-China, Northern China, Russia, Japan, and Korean Peninsula, as well as the homologous genes of *Medicago sativa* as the outgroup. The group of wild soybeans in Southern China is closer to *Medicago truncatula*; among them, a subgroup of Southern China wild soybean is very similar to that of *Medicago truncatula* (Fig. 2b). This result to some extent supports the assumption that wild soybean may be originated from low-latitude areas thereafter may spread to northward high latitude regions. However, more data are needed to support the origin of wild soybean, and more in-depth studies are needed to explore this important topic.

## Conclusions and future perspectives

**Fig. 3:**
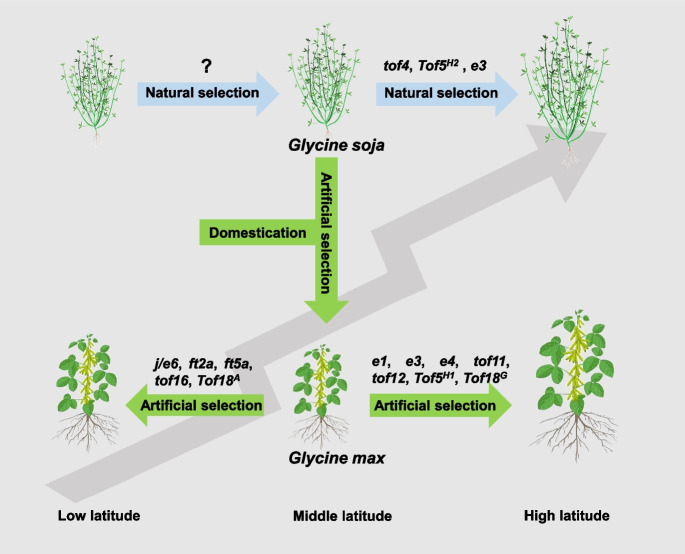
Different genetic mechanisms of latitudinal adaptation for wild (*Glycine soja*) and cultivated soybean (*Glycine max*)

Cultivated soybean and its wild ancestors are strict short-day plants and are highly sensitive to photoperiod. Like many crops, overcoming the limitations of the photoperiod sensitivity is crucial for the widespread adaptation of soybean to different environments. Over the course of domestication and improvement, new types of variants also appeared in cultivated soybean, and its sensitivity to light gradually decreased, making it become an important crop worldwide. There are many genes in soybean that helped it adapt to different latitudes which have undergone natural and artificial selection to enhance the adaptability of wild and cultivated soybean to different photoperiods (Fig. 3). When cultivated soybean expanded to low-latitude areas, the natural variants at *J*/*E6*, *Tof16*, *FT2a/LJ16.1*, and *FT5a/LJ16.2* conferring LJ traits and the allelic variants *Tof18*^*A*^ helped improve the adaptability of these plants to their new environments. Natural variation at *E1*, *E3*, *E4*, *Tof11*, and *Tof12* and allelic variation at some loci, such as *Tof5*^*H1*^ and *Tof18*^*G*^, have contributed to the adaptability of cultivated soybean to middle and high latitudes.

A thorough understanding of the genetic basis of photoperiod variation could facilitate the improvement of cultivated soybean and guide the breeding and production of this crop. In wild soybean, *Tof4* and *Tof5*^*H2*^ help regulate the plant response to photoperiod in middle and high-latitude regions. In addition, crop ancestors usually have rich allelic diversity, but most of these alleles were lost during crop domestication. Determining the genetic basis of the adaptation of wild soybean to different latitudes would help identify valuable genes or alleles from wild soybean. Natural variants from wild ancestors can be introduced into modern crops through breeding to improve plant adaptability and yield. Therefore, the discovery of genes related to the geographical adaptability of wild and cultivated soybean will help improve the flowering mechanism of soybean, promote the improvement of cultivated soybean, and provide an important theoretical and practical basis for molecular breeding of soybean varieties with enhanced adaptability and yields.

Photoperiod, the most stable environmental factor in nature, regulates many aspects of plant growth and development. Photoperiod not only affects the flowering time, maturity, and adaptability of soybean, but also affects seed growth and development and resistance to biotic and abiotic stresses, ultimately determining yield. Yu et al. (2023) demonstrated that three long-day plants (*Lotus japonicus*, *Pisum sativum*, and *Arabidopsis thaliana*) produce larger seeds under long-day conditions, whereas three short-day plants (*Glycine max*, *Vigna umbellata*, and *Phaseolus vulgaris*) produce larger seeds under short-day conditions; these traits are consistent with their photoperiodic flowering characteristics (Yu et al. 2023). Through in-depth research on the long-day plant Arabidopsis and the short-day plant soybean, the authors found that CO-AP2 functions in a maternally dependent manner by regulating the proliferation of epidermal cells in the seed coat, thus regulating seed size (Yu et al. 2023). The genes controlling the photoperiodic flowering of soybean also control resistance to high salinity and other stresses, including disease resistance. For example, the *LHY* homologs *LHY1a* and *LHY1b*, which encode components of the circadian clock in soybean, regulate plant responses to drought stress via abscisic acid signaling (Wang et al. 2021a). J promotes salt tolerance by upregulating the salt stress response-related genes *WRKY12*, *WRKY27*, *WRKY54*, *NAC*, and *SALT INDUCED NAC1* (*SIN1*) (Cheng et al. 2020). The loss of function of the circadian clock gene *E2* led to the upregulation of a peroxidase gene, enhancing the ability of soybean to eliminate reactive oxygen species under salt stress, thereby improving salt tolerance. Natural allelic variation leading to the loss of function of *E2* was often selected in soybean growing in high-latitude and high-saline-alkali areas (Dong et al. 2022b). *COL1a* enhances salt and drought resistance by promoting the accumulation of DELTA1-PYRROLINE-5-CARBOXYLATE SYNTHASE (P5CS), a major player in proline biosynthesis that functions in osmoregulation (Xu et al. 2023). Moreover, FT2a in aboveground tissue is a key factor determining the formation of soybean nodules (Li et al. 2022). FT2a moves from shoots to roots and activates the expression of the key nodule gene *EARLY NODULIN 40* (*ENOD40*) together with the transcription factor Nuclear Factor Y (NY-F) A-C, which is specifically responsive to low nitrogen levels, thereby inducing nodule formation and improving nitrogen use efficiency in soybean (Li et al. 2022; Wang et al. 2021b). Further investigations of photoperiod-regulated plant growth and development and stress tolerance will help us to understand the molecular mechanisms underlying the plant photoperiod responses.

In addition, soybean is also very sensitive to temperature changes. When soybean plants were exposed to high temperatures, they showed two opposite reactions in terms of flowering (Tang et al. 2023). Compared to the normal temperature of 25 °C, flowering was promoted at 30 °C but delayed at 35 °C. Late flowering at 35 °C occurred due to the regulation of *FT* expression by phyA-E1, whereas early flowering at 30 °C occurred due to the upregulation of *FT2a* and *FT5a* in a manner independent of *E1* (Tang et al. 2023). Beyond these explanations, why does soybean flower early at both low and high temperatures (30 °C) and late at even higher temperatures (35 °C)? What is the underlying molecular mechanism? How are photoperiod and temperature coordinated to regulate flowering? Further investigations are required to address these interesting questions.

Furthermore, what is the molecular mechanism underlying photoperiod sensitivity? Why do soybeans bloom early under short-day conditions and late under long-day conditions? While we know that *E1* is transcribed at low levels under short-day conditions and at high levels under long-day conditions (Xia et al. 2012), how is the transcription of *E1* regulated? How does this lead to differences in flowering under these conditions? How does photoperiod control the accumulation of quality, proteins, fats, isoflavones, and other nutrients? What is the effect of photoperiod on photosynthesis? The soybean growth period can be divided into two stages: the vegetative stages (flowering) and the reproductive stages (post-flowering). Timely flowering affects the yield of soybean, and post-flowering also plays an important role in the formation of seeds and yield. How can the vegetative and reproductive growth stages of soybean be reasonably controlled during the limited growth period to improve yields? The molecular mechanism underlying the initiation of soybean flowering is clearly understood, but studies of the late flowering stage have not been reported. These are all outstanding issues in soybean research.

## Data Availability

All data generated during the study are included in this published article.
